# From phase‐based to displacement‐based gating: a software tool to facilitate respiration‐gated radiation treatment

**DOI:** 10.1120/jacmp.v10i4.2982

**Published:** 2009-10-07

**Authors:** Joseph P. Santoro, Ellen Yorke, Karyn A. Goodman, Gig S. Mageras

**Affiliations:** ^1^ Department of Medical Physics Memorial Sloan Kettering Cancer Center New York NY USA; ^2^ Department of Radiation Oncology Memorial Sloan Kettering Cancer Center New York NY USA

**Keywords:** RPM gating, amplitude, phase

## Abstract

The Varian Real‐time Position Management (RPM) system allows respiratory gating based on either the phase or displacement (amplitude) of the breathing waveform. A problem in clinical application is that phase‐based gating, required for respiration‐correlated (4D‐CT) simulation, is not robust to irregular breathing patterns during treatment, and a widely used system version (1.6) does not provide an easy means to change from a phase‐based gate into an equivalent displacement‐based one. We report on the development and evaluation of a robust method to convert phase‐gate thresholds, set by the physician, into equivalent displacement‐gate thresholds to facilitate its clinical application to treatment. The software tool analyzes the respiration trace recorded during the 4D‐CT simulation, and determines a relationship between displacement and phase through a functional fit. The displacement gate thresholds are determined from an average of two values of this function, corresponding to the start and end thresholds of the original phase gate. The software tool was evaluated in two ways: first, whether in‐gate residual target motion and predicted treatment beam duty cycle are equivalent between displacement gating and phase gating during 4D‐CT simulation (using retrospective phase recalculation); second, whether residual motion is improved with displacement gating during treatment relative to phase gating (using real‐time phase calculation). Residual target motion was inferred from the respiration traces and quantified in terms of mean and standard deviation in‐gate displacement measured relative to the value at the start of the recorded trace. For retrospectively‐calculated breathing traces compared with real‐time calculated breathing traces, we evaluate the inaccuracies of real‐time phase calculation by measuring the phase gate position in each trace as well as the mean in‐gate displacement and standard deviation of the displacement. Retrospectively‐calculated data from ten patients were analyzed. The patient averaged in‐gate mean ± standard deviation displacement (representing residual motion) was reduced from 0.16±0.14cm for phase gating under simulation conditions to 0.12±0.08cm for displacement gating. Evaluation of respiration traces under treatment conditions (real‐time phase calculation) showed that the average displacement gate threshold results in a lower in‐gate mean and residual motion (variance) for all patients studied. The patient‐averaged in‐gate mean ± standard deviation displacement was reduced from 0.26±0.18cm for phase gating (under treatment conditions) to 0.15±0.09cm for displacement gating. Real‐time phase gating sometimes leads to gating on incorrect portions of the breathing cycle when the breathing trace is irregular. Displacement gating is less prone to such errors, as evidenced by the lower in‐gate residual motion in a large majority of cases.

In terms of duty cycle and residual motion, displacement‐based gating is equivalent to phase‐based gating for retrospectively‐calculated phase information.

PACS number: 87.55.ne, 87.59.cf, 87.90.+y

## I. INTRODUCTION

There is widespread use of respiration‐correlated CT, or 4DCT, for evaluating respiration‐induced tumor motion at simulation, defining treatment margins to account for motion, and selecting appropriate gate intervals for gated treatment.^(^ ^1^ ^–^ ^8^ ^)^ One such approach^(4)^ is to acquire repeat CT images over an entire respiratory cycle at each couch position (cine acquisition) while recording respiration with an external monitor of abdominal displacement (Real‐time Position Management RPM, Varian Medical Systems, Palo Alto CA). The CT images are then sorted according to respiration phase (Advantage 4D, GE Medical Systems, Waukesha, WI), to yield a series of volumetric CT images. The phase assigned to each image is calculated by a periodicity algorithm in RPM, where the 0% phase corresponds to end inhalation and approximately 50% to end exhalation. Since the images are tagged and sorted retrospectively, real‐time phase calculation is not necessary; thus RPM provides an option to retrospectively analyze the entire respiration trace in the phase calculation. At our institution, candidate patients for gated treatment receive a respiration‐correlated CT (RCCT) study at simulation, and the physician's choice of gate interval is based on the tumor motion observed in this study.

**Figure 1:**
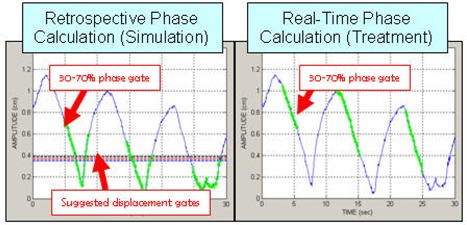
Retrospective phase calculation (left) where the green dots show a 30%–70% phase gate interval prescribed by the physician, and real‐time phase calculation (right) with 30%–70% gate shown in green; 0% phase is end‐inhalation (peaks in the trace), approximately 50% phase is end‐exhalation and the prescribed gate encompasses end‐exhalation.

The current capabilities of the RPM and CT scanner systems, however, pose a problem in their clinical application to gated treatment. The CT system correlates CT images only in terms of respiration phase, yet RPM performance in phase‐gated treatment is often unreliable with commonly encountered patient breathing patterns. The RPM system provides capabilities for gated treatment, using either phase‐based or displacement‐based mode. Phase‐based gated treatment requires real‐time calculation of the phase, which is thereby limited to analysis of the prior respiration trace up to the current instant in time. Dose delivery is enabled when the current phase of the trace lies between start and end phase values set by the user in a prior reference session. For some breathing patterns, real‐time calculation can intermittently assign the gating phase interval to an incorrect portion of the respiration trace; furthermore, this occurrence may be difficult for a therapist to identify. Figure 1 shows such an example, where the physician had chosen a gate interval of 30%–70% surrounding end‐exhalation at simulation (i.e. encompassing the respiration trace minima), but the real‐time phase calculation placed the gate interval much closer to end inhalation (trace maxima) than intended. The potential consequence is that the patient's internal anatomy is not at the intended position during the treatment gate and larger than intended residual motion occurs.

Displacement‐based gating is based directly on the current value of the respiration trace, rather than a quantity derived from its prior shape. Dose delivery is enabled when the respiration trace is between thresholds set by the user in a reference session. In a treatment session, the thresholds are automatically set relative to the minimum and maximum abdominal positions learned by the RPM system at the start of the session. In addition, the RPM graphic display for amplitude gating makes it easy for the therapist to see when irregularities cause the beam to be enabled at an incorrect part of the breathing cycle.

For the above reasons, clinical procedure at our institution is to deliver gated treatment in the displacement‐based mode. However, the CT system is not capable of displacement‐based gated simulation. Moreover, RPM version 1.6 does not provide a straightforward means to convert phase‐based gating interval (determined from the CT simulation) to an equivalent displacement‐based one. To address this, we acquire a displacement‐based reference session immediately after the RCCT simulation study for gated treatment purposes. This reference session is acquired within a few minutes of the patient's cine scan and is used for all subsequent treatment sessions. However, clinical workflow requires that the decision to set gate thresholds be postponed until hours or days later. Once that decision has been made, the physicist must determine the amplitude gate thresholds that best match the prescribed phase gate interval. The Varian RPM system allows either phase‐ or displacement (amplitude)‐ based gating, but changing a phase‐based gate to a displacement‐based gate (or vice versa) cannot be done within the same reference session. It can be a time‐consuming and inaccurate process if the only available tool is visual inspection of the “strip‐chart” type breathing traces provided by the RPM system.

To address this problem, we have developed a software tool that permits one to interactively analyze the phase‐based reference trace acquired during the cine‐CT session, and choose corresponding amplitude gate thresholds to apply to the treatment reference session.

## II. MATERIALS AND METHODS

To perform the study, we use a commercial system (Discovery ST, General Electric Medical Systems) which requires that an RPM phase‐based breathing trace be acquired simultaneously with a cine CT study. The RPM (Real‐time Position Management) system is an infrared video‐based system that monitors the position of a reflective marker block placed on the patient's abdomen.^(^ ^9^ ^–^ ^11^ ^)^ The patient receives audio coaching, customized to his/her breathing pattern, during the simulation in order to encourage regular breathing; the same patient‐specific instruction is used for gated treatment. The simulation software module (Advantage 4D) generates a respiration‐correlated CT image set at ten equispaced phase intervals using a retrospective recalculation of phases from the respiration trace.

**Figure 2:**
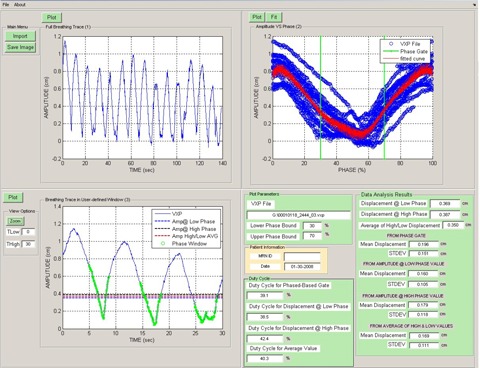
The respiratory trace analysis software with patient data (clockwise from the top left): (1) the entire breathing trace; (2) the displacement versus phase data (blue) with the fit (red) and phase gates (green); (3) plot of the displacement versus time trace with the phase gate (green circles) displacements at the low and high phase boundaries (blue and black dotted lines) as well as the average of these values (red dotted line); (4) a summary of the input file parameters and the results of the statistical analysis.

To facilitate the conversion of a phase‐based gating interval to a displacement (amplitude)‐based threshold, a custom software application was designed making use of the MATLAB (The Mathworks Inc., Natick, MA) software development toolkit. The software application parses the position, phase, and time information from a file containing the retrospectively phase‐calculated breathing trace recorded during the cine CT scan, and calculates displacements at each time point relative to the minimum (end exhalation) position recorded at the start of the session. The application uses the phase assignments from the trace to plot the displacement versus phase for each breathing period within the interval (0, 100%) such that the traces for all cycles are overlaid, permitting convenient visual inspection by allowing the user to evaluate the repeatability of the trace for the entire session at a glance. The application (Fig. 2) plots the displacement versus time of the respiration trace in the upper left panel, and displacement versus phase (blue circles) along with the physician‐specified phase gate interval (green vertical lines) in the upper right panel. The upper right plot is fitted to the following function (red curve in figure):

(1) $$A(P)=\sum_{i=1}^{8}A_{i}\;\text{sin}(B_{i}P+C_{i})$$

From this fit, one can extract a displacement *A* at any phase value *P*, where $A_{i},\;B_{i}$, and $C_{i}$ are fitted parameters, *i* is an index that ranges from 1 to 8. The choice of a linear combination of sine functions was made because it is both bounded and continuous, and eight terms were included to ensure that all shapes of breathing traces would be accommodated. From this fit, we determine the displacement from the end exhalation position at the start and end phases of the phase‐gate interval, as well as the average of these two values. The average value is often chosen as the displacement gate threshold, so this value will henceforth be emphasized. For evaluation purposes, the application also calculates the mean and standard deviation of the displacement within the displacement gate and the phase gate, as well as the predicted treatment beam duty cycles.

The lower left hand panel displays the breathing trace with the calculated displacement‐gate thresholds (dotted lines) and phase‐gate interval (green circles) overlaid on a plot. This window has functionality which allows the user to zoom to any portion of the breathing trace.

For the evaluation of the software tool, we evaluated two breathing traces from ten patients. The first trace, from the RCCT simulation in which the phase was retrospectively recalculated, tested whether the calculated displacement‐gated thresholds yielded similar in‐gate residual motion and duty cycle to the physician‐chosen phase‐gate thresholds, and the real‐time trace was used to demonstrate the lack of robustness in phase‐based gating. The second trace, representing gated treatment with real‐time phase calculation, tested whether residual motion was improved with displacement gating relative to phase gating. For 6 of 10 patients, the first trace was recorded on the same day as the second trace. These traces indicate patients who had both a phase‐based and an amplitude‐based simulation session recorded. For the remaining patients, the second trace was recorded twelve days to two months later. Residual target motion was inferred from the respiration traces and quantified in terms of mean and standard deviation in‐gate displacement. Average duration of the recorded traces was about 120 seconds.

## III. RESULTS

### A. Comparison of real‐time (prospective) vs. retrospective phase calculation

**Figure 3:**
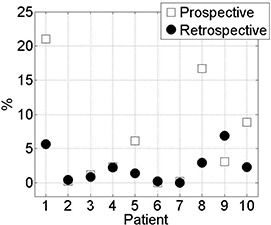
The fraction of time that the in‐gate patient breathing amplitude exceeded 30% of the maximum peak‐to‐peak amplitude for each patient. Black circles indicate a 30%–70% phase gate based on retrospective phase calculation; the white squares indicate the same gate and the same breathing trace using the real‐time (prospective) phase calculation.

Figure 3 shows the fraction of “gate on” time for which the mean in‐gate displacement exceeds 30% of the peak‐to‐peak displacement. Real‐time (prospective) phase calculation results in a higher fraction of large in‐gate displacements in four out of ten patients than retrospective phase gating. This comparison was made using a breathing trace from the same session. Large in‐gate displacements occurred up to 21% of the time in one patient. There is only one instance (Patient 9) where the percentage is less for real‐time phase calculation.

**Figure 4:**
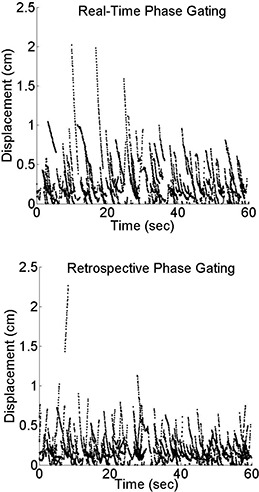
Displacement versus time for all patients using a 30%–70% gate with real‐time (prospective) phase calculation and retrospective calculation. The plots show only the first minute of each breathing trace.

Figure 4 shows scatter plots of in‐gate displacement (within the phase interval of 30%–70%) versus time, comparing real‐time phase (upper plot) and retrospective phase calculations (lower plot) for all ten patients. Each dotted curve segment (of typically 1 s duration) indicates the displacement versus time within a single “gate”. In retrospective phase calculation most displacements are below about 7 mm whereas in the real‐time phase calculation, a larger number of in‐gate displacements exceed this value, particularly at the start of each segment. This indicates that real‐time gating is often not centered on the intended end exhalation portion of the trace, but instead is positioned on the descending mid‐exhalation portion, similar to that illustrated in Fig. 1. Figures 3 and 4 indicate that real‐time phase calculation generally is less reliable than retrospective calculation.

### B. Comparison of displacement gating vs. retrospective phase calculation

**Figure 5:**
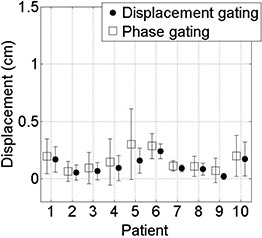
Mean in‐gate displacements from end exhalation at the start of session, and standard deviations (error bars) for (retrospective) phase‐based gate (square) and the average displacement‐based gate (circle) for simulation session data; the means and standard deviations (residual displacement) are larger for phase‐based gates than for displacement‐based gates in all patients.

**Figure 6:**
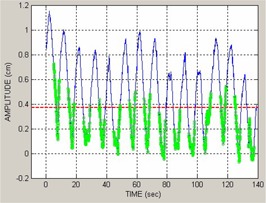
Breathing trace of Patient 1 showing the retrospectively calculated phase gate (green circles) and displacement gate from average of high and low phase points (red dotted line). The duty cycle from the displacement gate of this patient is 42% as compared with 39% for the phase gate.

We examine the equivalence of displacement gating using the proposed method and retrospective phase calculation in the simulation session data, in terms of in‐gate displacement and duty cycle. Figure 5 compares the mean and standard deviation in‐gate displacement for displacement gating versus retrospective phase gating in simulation data. The average displacement gate threshold results in a lower in‐gate mean and residual motion (variance) for all patients studied, which has been reported in other studies.^(^ ^12^ ^,^ ^13^ ^)^ Table 1 compares the duty cycle for displacement gating versus retrospective phase gating for simulation data. It can be seen that displacement gating yields a similar duty cycle to retrospective phase gating for simulation data. The duty cycle for the phase‐based gate is by construction 39% corresponding to the 30%–70% phase interval chosen by the physician. There is a slight asymmetry in the phase identification, characterized by an unequal distribution of the green phase points about the end‐exhalation minimum. This is caused by an overall irregularity of the typical breathing trace, characterized by random jitter or drift. This asymmetry effect is illustrated in Fig. 6. The green points represent the 30%–70% phase interval identified by the retrospective calculation. It is observed that the asymmetry of this phase interval about phase 50 (end exhalation) varies from cycle to cycle.

**Table 1:** Table shows the duty cycles for a displacement gate and for retrospective phase gating. The data is from a simulation session for each patient. The equivalence of displacement gating to retrospective phase gating is illustrated by the similarities in the duty cycle for each patient.

Patient	1	2	3	4	5	6	7	8	9	10
Displacement	42%	36%	43%	31%	36%	37%	34%	37%	36%	41%
Phase (retro)	39%	39%	39%	39%	39%	39%	39%	39%	39%	39%

### C. Comparison of displacement gating vs. real‐time gating

**Figure 7:**
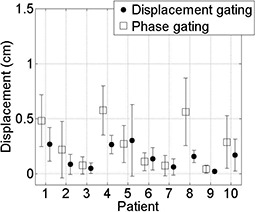
Mean in‐gate displacements from end exhalation at the start of session, and standard deviations (error bars) for real‐time phase‐based gate (square) and average displacement‐based gate (circle) for treatment session data; the means and standard deviations (residual displacement) are larger for phase‐based gates than for displacement‐based gates in 8/10 patients.

We compare displacement gating to real‐time (prospective) phase gating in Fig. 7 for each patient's second breathing trace (representing treatment session data), thus examining the anticipated performance of the two methods under gated treatment conditions. The mean in‐gate displacement and standard deviation are lower for displacement gating in eight out of ten patients compared to real‐time phase gating. The largest displacement with real‐time phase gating is 5.8 mm for Patient 4. The displacement gating reduces mean in‐gate displacement by a factor of 2 or more in two patients (Patients 4 and 8). The patient‐averaged mean ± standard deviation in‐gate displacement was reduced from 0.26±0.18cm for real‐time phase gating to 0.15±0.09cm for displacement gating.

## IV. DISCUSSION

The software tool allows the user to determine an appropriate displacement‐gated threshold in less than 5 minutes. This is a desirable feature since the RPM user is forced to choose between phase gating and displacement gating at simulation. A printout of the graphic display documents the patient's breathing performance at simulation as well as the calculation of the displacement‐gate threshold to be used for treatment. This method has proven robust even for irregular breathing patterns, although these cases are usually not treated with gating. For the fit function (Eq. 1), the index *i* was chosen by maximizing the *degrees of freedom adjusted R‐squared* statistic for Eq. 1 over the data. This statistic measures how successful the fit is in explaining the variation of the data. In other words, we plotted the DOF Adj R‐squared versus *i* for each patient for i=2 to 8. We observed that the DOF Adj R‐squared was maximized at i=8 for 9 out of 10 patients. Increasing *i* beyond 8 significantly slowed down the processing speed needed to complete the fit without the added benefit of higher quality.

The RPM real‐time phase calculation algorithm has been observed to miscalculate the phase positions, relative to the respiration trace, during the treatment session for some patients, resulting in an unintended phase gate during treatment. Therefore, we currently use displacement‐based gating for treatment. We acquire a short displacement‐gated reference session during the patient's simulation session and use the software tool described here to guide the choice of displacement window that agrees with the physician's chosen phase gate. The major benefits of the software are that it allows the user to (1) quickly make an informed decision about amplitude gate based on an analysis of the respiration trace, and (2) evaluate the patient's breathing pattern at simulation in terms of statistics (mean and standard deviation) of the in‐gate displacement and the estimated treatment beam duty cycles for phase and displacement gating, in order to decide whether gated treatment is appropriate.

Other investigators have examined the RPM system for 4DCT and treatment. Vedam et al.^(14)^ determined a way of generating a real‐time (prospective) displacement gate for radiation delivery from the more reliable retrospective‐determined phase information. In their method, a displacement gate threshold is obtained from the breathing trace at simulation, based on the average and maximum respiratory displacement within the phase gate interval. The displacement threshold to be used for treatment is determined iteratively from simulation data. Day‐to‐day variations can occur, not only in motion of the external marker surrogate but also in internal anatomy, even in the presence of consistent external motion.^(15)^ These and similar findings, together with the advent of kV‐based image guidance (Varian OBI) have changed our clinical practice regarding respiratory gating. Preferred patients for gated treatment are those with radio‐opaque objects (stents, surgical clips or fiducial markers) near or in the tumor that serve as surrogates for target position. These are displayed on orthogonal DRRs with a margin corresponding to their motion within the (phase) gate observed on the 4DCT. The position of the surrogates are checked daily prior to (displacement gated) treatment by registering to gated kV radiographs, while motion extent is checked less frequently (to limit the imaging dose) by “gated” fluoroscopy.

Baseline drift has a different effect on the software depending on the type of gating. In displacement gating, a baseline drift that is comparable to or larger than the separation between gating thresholds (often about one‐third the peak‐to‐trough amplitude of the trace) will cause the breathing trace to drift out of the thresholds and the machine will no longer gate on end expiration. This situation is easily detected by the therapist. In phase gating, a baseline drift per cycle of less than 10%–20% of the breathing amplitude (depending on the threshold of the normal breathing predictive filter) will be ignored by the software. Thus a potentially large baseline drift can accumulate after a few cycles which is not readily apparent to the therapist. Ruan et al.^(16)^ have described a method of correcting for baseline drift, in which the mean position of motion is determined from the respiration trace without explicitly estimating instantaneous phase. Although the method is shown to be robust, it requires real‐time access and analysis of the respiration trace during treatment, which is currently not possible with the RPM system. (RPM allows saving of the data to a file only after treatment delivery has stopped.)

Mutaf et al.^(17)^ have shown that errors in the phase assignment during 4DCT can lead to artifacts in the phase‐sorted images (i.e. discontinuities in the anatomy between consecutive CT slices). They found that phase calculation errors occurring during phase‐gated treatment may lead to inaccuracies in structure localization and target delineation in treatment planning. Our method is based on a functional fit to the retrospectively determined displacement‐phase data to determine the relationship between a phase gate at simulation and displacement gate used for treatment.

## V. CONCLUSIONS

Real‐time phase gating often does not correctly determine the desired portion of the breathing trace under commonly encountered breathing conditions. The proposed method of determining thresholds for displacement‐based gating yields similar in‐gate displacement, residual motion, and duty cycle to those established from analysis of respiration‐correlated CT at simulation using retrospective phase calculation. Displacement gating reduces in‐gate displacement and residual motion relative to real‐time phase gating under gated treatment conditions.

Our findings suggest that the displacement gate method proposed here reduces residual breathing motion during treatment relative to phase‐gated treatment, as evaluated by the lower mean in‐gate displacement and residual motion for eight out of ten patients. This can potentially translate into less internal target motion. Further validation in a future study will use fluoroscopic imaging in the treatment room to quantify in‐gate displacement of an implanted fiducial marker.

## ACKNOWLEDGEMENTS

This work was supported in part by Award Numbers P01‐CA59017 and T32‐CA61801 from the National Cancer Institute, and by a research grant from Varian Medical Systems. The content is solely the responsibility of the authors and does not necessarily represent the official views of the National Cancer Institute or the National Institutes of Health.
